# Improved Slow Light Capacity In Graphene-based Waveguide

**DOI:** 10.1038/srep15335

**Published:** 2015-10-19

**Authors:** Ran Hao, Xi-Liang Peng, Er-Ping Li, Yang Xu, Jia-Min Jin, Xian-Min Zhang, Hong-Sheng Chen

**Affiliations:** 1 College of Information Science and Electronic Engineering, Zhejiang University, Hangzhou 310027, Zhejiang, China

## Abstract

We have systematically investigated the wideband slow light in two-dimensional material graphene, revealing that graphene exhibits much larger slow light capability than other materials. The slow light performances including material dispersion, bandwidth, dynamic control ability, delay-bandwidth product, propagation loss, and group-velocity dispersion are studied, proving graphene exhibits significant advantages in these performances. A large delay-bandwidth product has been obtained in a simple yet functional grating waveguide with slow down factor *c*/*v*_*g*_ at 163 and slow light bandwidth Δω at 94.4 nm centered at 10.38 μm, which is several orders of magnitude larger than previous results. Physical explanation of the enhanced slow light in graphene is given. Our results indicate graphene is an excellent platform for slow light applications, promoting various future slow light devices based on graphene.

In recent years, slow light has attracted extensive attentions due to its potential for optical memory which is one of the most challenging components^1^ in optical integrated circuits. Optical signal in a media with refractive index *n* has a light speed of 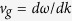, whereas the slow-down factor^2^ (S factor) which describes how many time light has been slowed is defined as:


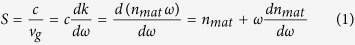


the S factor is strongly dependent on two issues: the material index *n*_*mat*_ and the material dispersion *dn*_*mat*_/*dω*. According to Eq. (1), large S factor can be achieved in the materials whose *n*_*mat*_ is large or whose dispersion *dn*_*mat*_/*dω* is large. However, the largest material index available in nature is only 4 for germanium^3^, indicating that the slow light capabilities of natural materials are poor^4^. On the other hand, the material dispersion is usually small in conventional materials. The material dispersion increases near the index resonances that rarely occur at resonance frequencies of noble metals such as gold and silver, where absorption becomes a problem.

Novel materials are required to improve the slow light capacity^5^. Recently the two-dimensional (2D) material graphene has shown unprecedented ability to enhance the light-matter interaction^5^,^6^. Especially, a large material index of 69.34 has been reported in graphene which is more than one order of magnitude larger than other materials^7^. Ref. 8 disclosed an extremely large dispersion in well-designed graphene sheet, revealing the possible dispersion engineering in graphene. It is therefore inferred that graphene has promising potential in slow light. In addition, the large actively-control of graphene’s permittivity^9^ may enable freedom to control the slow light performances during operation that has not been seen in natural materials. The one-atomic thickness of graphene has intrinsic advantage to minimize the footprint of slow light devices^10^. However, until now a clear physical understanding and a systematic investigation of the slow light capability in graphene have not been fully explored.

In this paper, a comprehensive study of slow light propagation on graphene is performed, including material index, slow light bandwidth, dynamic control ability, delay-bandwidth product, propagation loss, and group-velocity dispersion. It can be concluded that graphene has shown significant advantages in all these aspects. A largely improved delay-bandwidth product has been obtained after optimizations with S factor of 163 and slow light bandwidth Δω of 94.4 nm, which is rarely seen in other materials. Physical understanding of the improved slow light capability is further explained. It is concluded that graphene exhibits large slow light capability, therefore can serve as an excellent slow light platform.

## Results and Discussion

### Large material dispersion

**Figure 1:**
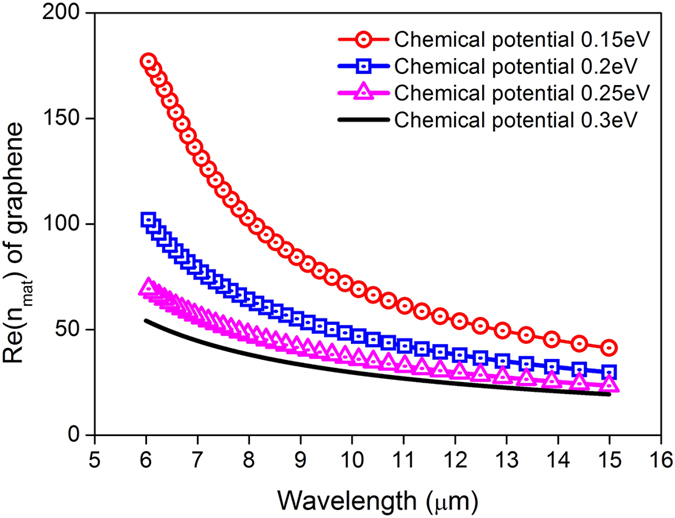
The real part of graphene’s material index varies with chemical potential and wavelength.

Owing to the one atomic layer thickness, graphene’s material properties are highly tunable with external environmental parameters such as temperature, chemical potential, scattering rate^7^,^8^,^9^, etc. The surface conductivity can be modelled following the Kubo formula^11^, from which the surface (2D) permittivity can be derived. For convenience, in Fig. 1 the effective material index (*n*_*mat*_) is calculated from the surface permittivity. The real part of *n*_*mat*_ shows monotonic decrease if the considered wavelength increases from 6 μm to 15 μm. *n*_*mat*_ also decreases with the increase of chemical potential. For small wavelength and small chemical potential, the material index values are high, e. g. for wavelength at 6 μm, *n*_*mat*_ is several orders of magnitude larger if compared with natural materials. This is in accordance with Vakil and Engheta’s observation that freestanding graphene exhibits a high refractive index of 69.34^7^ (called large material dispersion of graphene). Moreover, because *ω* > 0 and *dn*_*mat*_/*dω* > 0, in Eq.(1) the S factor of graphene is proportion to its material index. As the material’s indices in Fig. 1 are large, the S factors of graphene are then expected to be large accordingly, e. g. at chemical potential of 0.15 eV and wavelength around 6 μm, the S factor is expected to be larger than 170. The extreme large values of *n*_*mat*_ in Fig. 1 indicate that light speeds in graphene can be slowed intensively, where S factors are much larger than conventional materials.

### Structural dispersion

**Figure 2:**
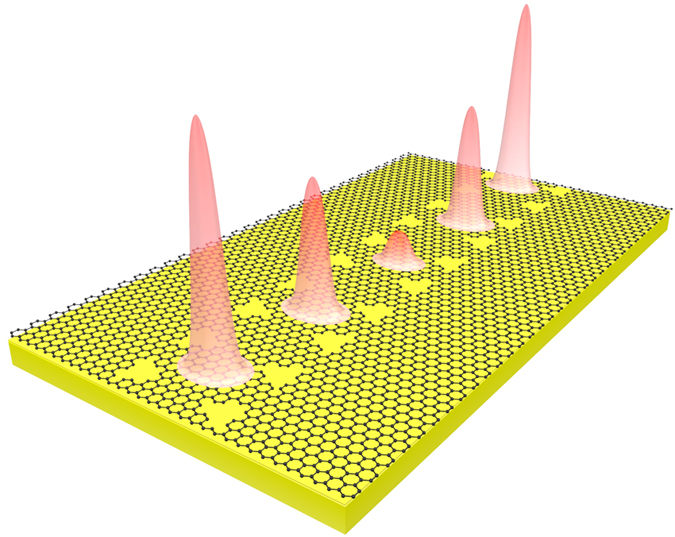
The schematic picture of the proposed slow light waveguide.

Previous knowledge in photonic crystals has suggested that periodical repeated patterns are able to enlarge the dispersion^2^. E. g. A periodically repeated ring-shaped air holes was used in photonic crystal waveguide to engineer the dispersion curve^12^; the position of air holes has been shift to obtain a U type dispersion curve which improved the overall delay-bandwidth product^13^; Chen *et al.* developed a graded-grating-loaded plasmonic waveguide for slow waves in telecom and infrared frequencies^14^, etc. In all these studies, periodical repeated patterns have shown strong influences to the structure’s response to different frequencies, which is highly related to the second item 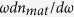 in the right side of Eq. (1)^15^. Following this methodology, here two lines of triangle-shaped air holes are periodically added on the surface of the graphene sheet, as depicted in Fig. 2. The length of the horizontal side in triangle is 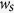 = 1000 nm, the periodicity for the grating is chosen to be P = 1205 nm, and the gap between the two lines of air holes is 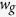 = 400 nm. To facilitate the simulation, a freestanding graphene with 2D effective index methodology^7^ is considered here. Light is incident into the grating along the gap between the two lines of air holes.

**Figure 3:**
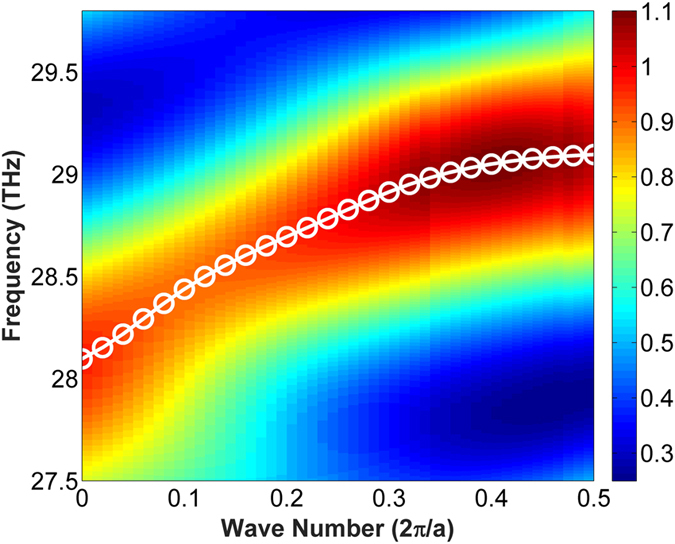
The dispersion curves of the proposed waveguide under the chemical potential at 0.2 eV. The white circle curve indicates the waveguide modes. The length of horizontal side in triangle hole (*w*_*s*_), period (P), waveguide width (*w*_*g*_) for the waveguide are chosen to be *w*_*s*_ = 1000 nm, P = 1205 nm, *w*_*g*_ = 400 nm, respectively.

In Fig. 3, the electric energy distribution of the potential modes in frequency domain are plotted as colorful contour map. By searching the maximum electric energy in the spectrum, the high intensity energy peaks are marked as while circle line in Fig. 3, indicating the existence of the intrinsic guiding modes^8^,^16^. The dispersion curve which records the relation between frequency
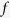
and wave number 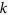 are then obtained. The details of energy spectrum calculation can be found in method. The obtained guiding modes in Fig. 3 not only ensure the confinement of light but also provide the single mode propagation. Furthermore, it can be seen clearly that the dispersion curve is made up of two high intensity energy peak regions: one is k approaching 0, and the other is k close to band edge. This is in accordance with previous finding^15^ that light is conducted under two mechanisms in periodical structures: the index-guiding mechanism where *k*_*x*_ approaches 0 and the gap-guiding mechanism where *k*_*x*_ is close to the band edge. The conjunction of the two parts are affected by the inserted air holes.

**Figure 4:**
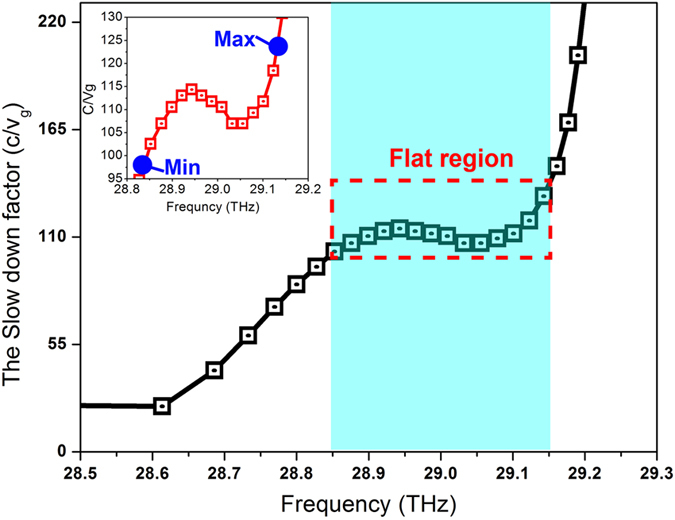
The slow-down factor curve varies with Frequency. The length of horizontal side in triangle hole (*w*_*s*_) is chosen to be *w*_*s*_ = 1000 nm, and the waveguide width *w*_*g*_ = 400 nm.

The ratio of the dispersion curve (denoted as S factor) is depicted in Fig. 4, where the S factor curve exhibits a decrease-constant-decrease curve shape, called “stair-like” slow light^17^. One significant feature is that a large flat region is observed in the middle of the S factor curve, which is apparently different with previous slow light publications in graphene^5^,^8^,^18^. The flat region in the curve ensures a wide band slow light operation in Fig. 4, where different frequency components inside the bandwidth can be delayed to the same speed. This is very important for slow light devices because different light speeds between different frequencies may cause seriously signal distortions that affect the slow light performances. To rigorous investigate the flat feature, the concept of flat ratio is used^13^, defined as the 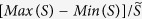, where 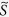 is the average value, as depicted in the insert picture of Fig. 4. If we regard 10% as flat S factor threshold, which is only half value to ref. 13, the obtained slow light bandwidth is 89.6 nm under S factor centered at 110.3 and bandwidth centered at 29 THz. Furthermore, the dispersion curve in Fig. 3 and the S factor curve in Fig. 4 are compared. In Fig. 3, a linear flat dispersion curve can be observed for the frequencies around 29 THz where the curve inside the bandwidth maintains the same ratio. This corresponds to the constant S factor of 110.3 in Fig. 4 for frequencies from 28.86 THz to 29.15 THz. Rigorous calculation shows a large slow light bandwidth of 89.6 nm can be achieved. It should be mentioned large S factor usually leads to limited bandwidth^17^. As a reference, previously the reported bandwidth is only 2.4 nm for S factor around 96 in a well-designed photonic crystal waveguide^13^. The significantly increased slow light bandwidth in graphene reveals that the slow light capability has been improved.

Let’s take a deeper look into the physical understanding of the improved slow light capability in graphene. The material dispersions in natural available materials are low. Therefore, previous researchers try to enlarge the dispersion by well-designed periodical structures. However, the new material graphene is demonstrated to provide several orders of magnitude larger material index than other materials, thus a much larger material dispersion can be obtained by the material itself, which is facilitate to slow down the light speed. In this paper, we used graphene as slow light material to obtain large material dispersion, and simultaneously designed periodical structure to enlarge the structural dispersion. Such arrangements ensure the designed device can benefit from both large material dispersion and large structural dispersion, both of which can contribute to the improvement of slow light capacity.

### The dynamical control ability

One of the major advantages of graphene is its large active-controlled permittivity under the external environment parameter variations, such as temperature T, charged particle scattering rate Γ, angle frequency ω, and chemical potential μ. Among these parameters, the chemical potential μ in graphene is directly linked to the external applied gate-voltage that provides a convenient approach to control graphene’s permittivity. The chemical potential is defined as:


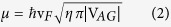


where V_*AG*_ = *V*_*g*_ − *V*_*Dirac*_ is the external applied voltage, *V*_*F*_ is the Fermi velocity, and *η* = 9 × 10^16^*m*^*−*2^*v^−1^* 19. The direct control of graphene’s chemical potential provides a flexible but convenient way to tune the slow light performance: By applying different external voltages, graphene’s permittivity can be changed, thus the slow light performances can be influenced. The emerging of graphene with slow light waveguide has provided an unprecedented way to dynamically control the performances.

**Figure 5:**
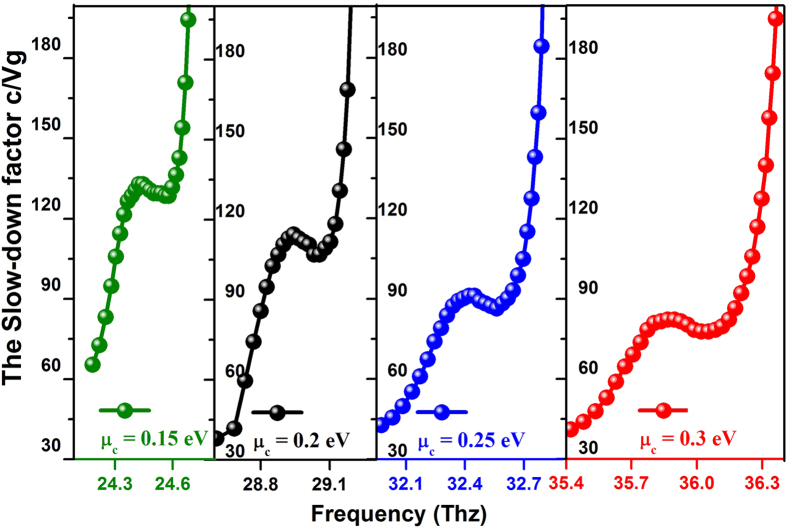
The slow-down factor varies with the frequencies at chemical potentials: (a) *μ*_*C*_ = 0.15 eV, (b) *μ*_*C*_ = 0.2 eV, (c) *μ*_*C*_ = 0.25 eV, (d) *μ*_*C*_ = 0.3 eV.

Figure 5 shows the S factor curves under different chemical potentials. The shift of the chemical potential does not affect the stair-like slope of the curves but influences the constant S factor value. When the chemical potential is gradually increased from 0.15 eV to 0.3 eV, the constant S factor decreased from 130 to 80. This is expected, because the permittivity of graphene decreases when the chemical potential increases, resulting in the decrease of the dispersion curve ratio (the S factor). On the other hand, the slow light bandwidth for constant S factor moves to higher frequency and the bandwidth increases with chemical potential.

We would like to highlight the convenience of dynamic control ability via graphene sheet. The slow light performances including the S factor, bandwidth, frequency can be modified by the external applied voltage. Please note that the control method via graphene here can be implemented during the slow light operation without re-fabrication or modification of the device, which is quite different from previous slow light controls^20^,^21^.

### The delay-bandwidth product

Although the S factor 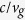 represents the degree of slow down, it is not a full picture of slow light performances. There exists a compromise between the S factor and the corresponding slow light bandwidth. Neither emphasizing S factor nor bandwidth is unfair, e. g. a large S factor but narrow bandwidth pulse would causes signal distortion. Therefore, the concept of normalized delay-bandwidth product (NDBP) is introduced as an overall parameter to estimate the slow light performances^17^ (see methods).

**Figure 6:**
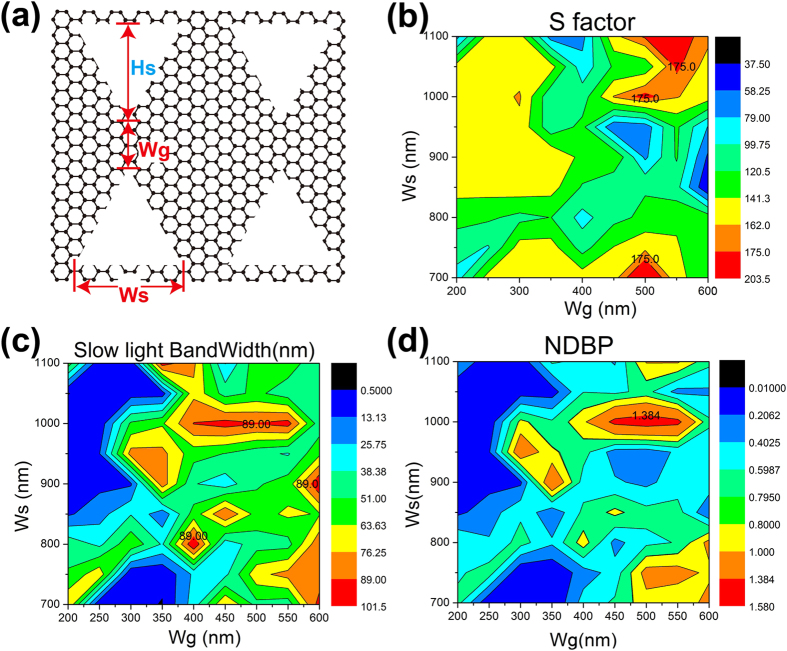
The full geometrical optimization of the slow light performance. we set 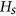 at 680 nm, while sweeping *w*_*s*_ and *w*_*g*_ simultaneously. (**a**) the schematic picture for the three geometrical parameters, the optimization results for: (**b**) the S factor, (**c**) the slow light bandwidth, (**d**) NDBP.

Here we take a full sweep of the geometrical parameters including the waveguide width *w*_*g*_, the height *H*_*s*_ and the width 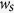 of the holes (see Fig. 6(a)). Additionally, since the ratio between *H*_*s*_ and 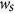 represents the zenith angle of the triangular hole, we only need to consider one of them. In the following discussion, we set *H*_*s*_ at 680 nm, while sweeping *w*_*s*_ and *w*_*g*_ simultaneously. The triangular width *w*_*s*_ is taken from 700 nm to 1100 nm with an increasing step of 50 nm, while the waveguide width *w*_*g*_ is taken from 200 nm to 600 nm with an increasing step of 50 nm. The obtained slow down factor, slow light bandwidth, and NDBP are shown in Fig. 6(b–d), respectively. All the slow light bandwidth and NDBP are obtained under flat ratio 10% which is a strict constraint if compared with previous publications^13^,^15^,^16^,^20^. Because not all considered parameters have stair-like S factor curves, the optimization criterion here is: If there is stair-like S factor curve, the S factor and bandwidth are calculated from the flat region as depicted in Fig. 4; If there is no stair-like curve but a monotonically increasing curve, we fixed the S factor at 150 and calculate the corresponding bandwidth with the restrain of flat ratio 10%. As can be seen in Fig. 6(b), there are three regions for large S factor >175, which have common feature that *w*_*g*_ range from 500 nm to 550 nm. However, the optimizations show three maximum slow light bandwidth Δ*ω* regions in Fig. 6(c), one is around (*w*_*g*_, *w*_*s*_) centered at (400 nm, 800 nm), one is around (*w*_*g*_, *w*_*s*_) centered at (600 nm, 900 nm), the other one is *w*_*g*_ from 400 nm to 550 nm and 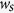 around 1000 nm. These three regions show maximum bandwidth Δ*ω* larger than 89 nm which is extremely large value than previous results^13^,^15^,^16^,^20^,^21^. It should be noted that there exist an overlap region for (*w*_*g*_,*w*_*s*_) centered at (500 nm, 1000 nm) where both S factor and bandwidth are large, which indicates a large NDBP value. As the NDBP is the product of 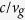 and Δ*ω*, NDBP should be the compound results from both 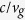 and Δ*ω*. However, interestingly, we found the optimization result of NDBP in Fig. 6(d) is similar with the Δ*ω* result in Fig. 6(c), while the S factor in Fig. 6(b) has few influence to the final NDBP result. It is then inferred that the bandwidth has dominated contribution of configuring NDBP. In Fig. 6(d), the largest NDBP value 1.48 occurs at 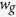 = 550 nm and 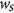 = 1000 nm, where the S factor is 163 and Δ*ω* is 94.4 nm.

### The Loss and the GVD

**Figure 7:**
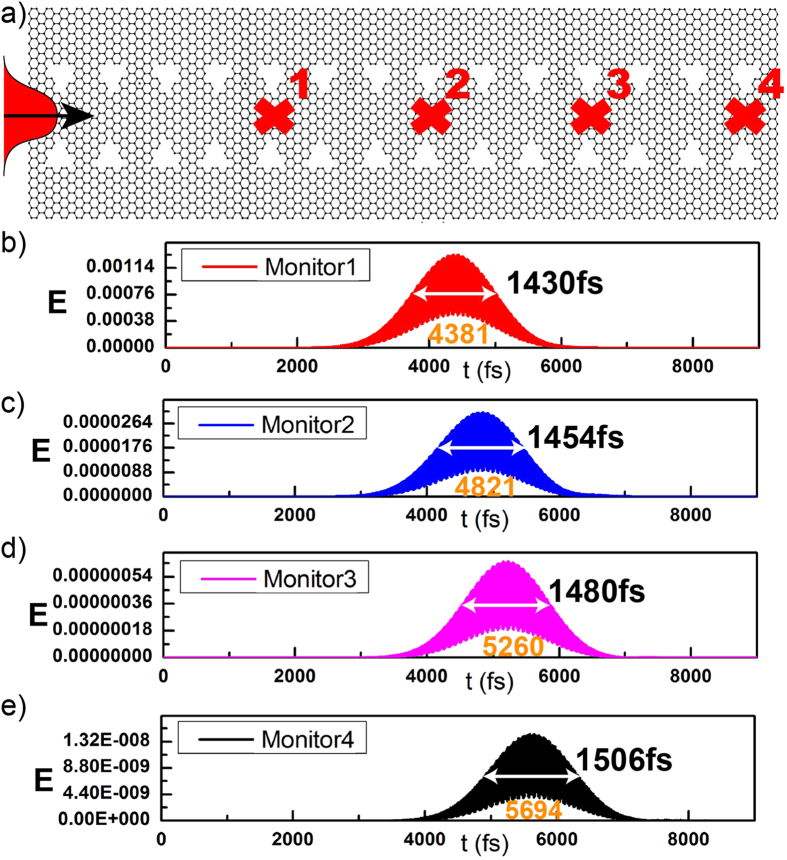
(**a**) The schematic picture of the pulse simulation, and the real part of electric field with time evaluation at (**b**) monitor 1, (**c**) monitor 2, (**d**) monitor 3, (**e**) monitor 4 along the propagation direction when the chemical potential μ_C_ at 0.2 eV.

Rigorous simulations are used here to estimate the propagation loss in the waveguide. A Gaussian pulse is injected into the waveguide with *w*_*g*_ of 400 nm and chemical potential μ of 0.2 eV, as depicted in Fig. 7(a). From previous band calculation results in Fig. 4, a wide flat bandwidth from 28.9 THz to 29.1 THz has been observed under these parameters. Thus here we set the Gaussian pulse centered at 29 THz with a width of 0.2 THz. The source is located 1000 nm away from the waveguide to avoid the injection perturbation. Four time monitors are added along the propagation direction with an interval distance of 1205 nm. Loss can be estimated by compare the peak values of optical energies. The ratio of peak energies between monitor 2 and 1, monitor 3 and 2, monitor 4 and 3 are −27.1 dB, −27.8 dB and −27.3 dB, respectively. Before we make further comment on the loss, the S factor can also be estimated in Fig. 7 by counting the envelope’s peak movement. The time delay between monitor 2 and 1, monitor 3 and 2, monitor 4 and 3 are 440 fs, 440 fs and 434 fs, respectively, which corresponds to the same S factor of 109.8 between the adjacent monitors. The pulse result of S factor at 109.8 is in good accordance with previous result of S factor of 110.3 from band calculation in Fig. 4, which proves the accuracy of the simulation. It should be pointed out that the obtained loss values here are not high, if consider they are retrieved under the large S factor of 110. Because the slow light loss is proportional to the corresponding S factor^20^,^22^. E. g., as a comparison, ref. 20 records a loss around 30 dB in silicon-based grating waveguide under the same S factor 110. Smaller loss can be achieved if shitting the S factor to smaller values, which can be done in terms of increasing the chemical potential in Fig. 5.

Group velocity dispersion (GVD) is the phenomenon that the group velocity of light in a medium highly depends on the optical frequency. A simple way to evaluate the GVD parameter of the proposed waveguide can also be derived from Fig. 7. The GVD parameter can be estimated by measuring the broadening of the pulse. The waist of the pulse during the pulse propagation can be quantified by the Full-Width Half-Maximum (FWHM) value. In our simulation, the FWHM of the pulses at monitor 1, 2, 3 and 4 are 1430 fs, 1454 fs, 1480 fs, and 1506 fs, respectively. Thus the pulse broadening between monitor 2 and 1, monitor 3 and 2, monitor 4 and 3 are 24 fs, 26 fs and 26 fs, respectively. As a consequence, it can be concluded that the pulse with a waist length around 1430 fs has been broadened by only 25 fs after propagating 1.205 μm distance. Taking the slow light bandwidth 89.6 nm into account, this means the broadening of the pulse is only 0.32 fs per nanometer wavelength change and per micrometer propagation distance. This corresponds to a GVD parameter of 0.32 fs·nm^−1^·μm^−1^, which is an extremely low GVD value if compared with previous results^12^,^23^,^24^,^25^, e. g. the obtained GVD is two orders of magnitude smaller than ref. 25, and one order of magnitude smaller than ref. 12. It should be pointed out that this ultra-low GVD is retrieved when the S factor is large at 110 and corresponding bandwidth is large at 89.6 nm.

## Conclusion

In summary, we employed 2D material graphene to slow down and actively control the light speed. Systematical investigations of slow light performances were performed. By optimizing the material dispersion and structural dispersion, the improved slow light capability has been obtained and the results showed an extremely large NDBP value with *c*/*v*_*g*_ at 163 and Δ*ω* at 94.4 nm, which is larger than previous results. The dynamical control of the wide band slow light with ultra-low GVD effect has been demonstrated. It can be concluded that graphene exhibits significant advantages in all the figure of merits in slow light, showing great potential in future light memories and optical integrated circuits.

## Methods

### Material modeling

It was theoretically demonstrated and experimentally verified that, graphene’s conductivity can be modeled following the Kubo formula as below:


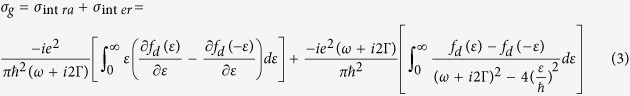


where *f*_*d*_(*ε*) is the Fermi function, *e* is the electric charge, 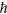 is the plank constant, *ω* is the angular frequency, and Γ is the scattering rate. When σ_*g*_ > 0, the whole graphene sheet acts like a thin metallic layer which facilitates the propagation of surface plasmon polaritons. The 2D permittivity of graphene can be obtained from the dispersion relation of SPP below:


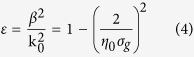


Where β is the propagation constant, *k*_0_ is the wavenumber in vacuum, *η*_0_ is the intrinsic impedance of free space. The material’s refractive index *n*_*mat*_ can be estimated via 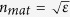.

### Dispersion curve calculation

The dispersion curves are calculated from a special technology from rigorous Finite Difference Time Domain method. In the calculation, one unit cell of the designed periodical structure is considered where the Bloch boundary condition is applied in the propagation direction and the perfectly matched layer absorbing boundary condition is applied in the other direction. We placed one randomly phased dipole source in the optimized position (where the intrinsic modal energy is maximum) to excite the waveguide mode as strong as possible. 10 monitors are randomly placed inside the unit cell to record the electromagnetic wave evolution with time. Symmetrical condition can be used to accelerate the calculation according to the polarization state. After running simulation with sufficient time, some of the excited modes will decay which is the inappropriate modes for the structure (the fake modes), and the modes let in the simulation region are the intrinsic modes of the structure. The fields in the time monitors are then collected and transferred to the frequency domain by appropriate Fourier transformation. To get accurate spectrum in frequency domain, the time domain data need to be apodization filtering to isolate the instant response of the light source. The frequency domain energy spectrum (the colorful contour map in Fig. 3) is obtained by adding the Fourier transformation of all the time monitors together, then the energy peaks in the spectrum identifies the intrinsic mode (contain the information of eigen-frequency *f* and wavenumber *k*). By sweeping the wavenumber value *k* as input, we obtained the full information of the frequency domain energy spectrum. The dispersion curve can be derived by searching the energy peak values of eigen-frequency *f* under each *k* in the frequency domain energy spectrum (the white circle line in Fig. 3).

### Delay-bandwidth product calculation

Delay-bandwidth product contains the information of both delay and bandwidth, thus it is a relatively fair way to evaluate the slow light performance. Its normalized form, the normalized delay-bandwidth product (NDBP) is more frequently used, defined by:


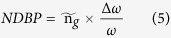


The average group index 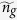 is calculated by:


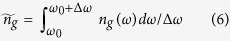


## Additional Information

**How to cite this article**: Hao, R. *et al.* Improved Slow Light Capacity In Graphene-based Waveguide. *Sci. Rep.* **5**, 15335; doi: 10.1038/srep15335 (2015).

## References

[b1] Yanik M. F. & Fan S. Slow light: Dynamic photon storage. Nat. Phys. 3, 372–374 (2007).

[b2] Krauss T. F. Slow light in photonic crystal waveguides. J. Phys. Appl. Phys. 40, 2666 (2007).

[b3] De La Rue R. M. Optical delays: Slower for longer. Nat. Photonics 2, 715–716 (2008).

[b4] He S., He Y. & Jin Y. Revealing the truth about ‘trapped rainbow’ storage of light in metamaterials. Sci. Rep. 2, 583 1:9 (2012) 10.1038/srep00583. 22900141 PMC3419922

[b5] Lu H. *et al.* Graphene-based active slow surface plasmon polaritons. Sci. Rep. 5, 8443 1:7 (2015) (10.1038/srep08443). 10.1038/srep08443 PMC4327412 25676462

[b6] Koppens F. H. L., Chang D. E. & García de Abajo F. J. Graphene Plasmonics: A Platform for Strong Light–Matter Interactions. Nano Lett. 11, 3370–3377 (2011). 21766812 10.1021/nl201771h

[b7] Vakil A. & Engheta N. Transformation Optics Using Graphene. Science 332, 1291–1294 (2011). 21659598 10.1126/science.1202691

[b8] Hao R., Jin J., Peng X. & Li E. Dynamic control of wideband slow wave in graphene based waveguides. Opt. Lett. 39, 3094–3097 (2014). 24875985 10.1364/OL.39.003094

[b9] Ooi K. J. A., Chu H. S., Ang L. K. & Bai P. Mid-infrared active graphene nanoribbon plasmonic waveguide devices. J. Opt. Soc. Am. B 30, 3111 (2013).

[b10] Brar V. W., Jang M. S., Sherrott M., Lopez J. J. & Atwater H. A. Highly Confined Tunable Mid-Infrared Plasmonics in Graphene Nanoresonators. Nano Lett. 13, 2541–2547 (2013). 23621616 10.1021/nl400601c

[b11] Hanson G. W. Dyadic Green’s functions and guided surface waves for a surface conductivity model of graphene. J. Appl. Phys. 103, 064302 (2008).

[b12] Säynätjoki A., Mulot M., Ahopelto J. & Lipsanen H. Dispersion engineering of photonic crystal waveguides with ring-shapedholes. Opt. Express 15, 8323–8328 (2007). 19547162 10.1364/oe.15.008323

[b13] Hao R. *et al.* Novel slow light waveguide with controllable delay-bandwidth product and utra-low dispersion. Opt. Express 18, 5942–5950 (2010). 20389613 10.1364/OE.18.005942

[b14] Chen L. *et al.* Broadband slow-light in graded-grating-loaded plasmonic waveguides at telecom frequencies. Appl. Phys. B 104, 653–657 (2011).

[b15] Hao R. *et al.* Improvement of delay-bandwidth product in photonic crystal slow-light waveguides. Opt. Express 18, 16309–16319 (2010). 20721017 10.1364/OE.18.016309

[b16] Xu Y., Zhang J. & Song G. Slow Surface Plasmons in Plasmonic Grating Waveguide. IEEE Photonics Technol. Lett. 25, 410–413 (2013).

[b17] Baba T. Slow light in photonic crystals. Nat. Photonics 2, 465–473 (2008).

[b18] Zhou H. *et al.* Enhanced four-wave mixing in graphene-silicon slow-light photonic crystal waveguides. Appl. Phys. Lett. 105, 091111 (2014).

[b19] Hao R. *et al.* Ultra-compact optical modulator by graphene induced electro-refraction effect. Appl. Phys. Lett. 103, 061116 (2013).

[b20] Casas-Bedoya A. *et al.* Slow-light dispersion engineering of photonic crystal waveguides using selective microfluidic infiltration. Opt. Lett. 37, 4215–4217 (2012). 23073415 10.1364/OL.37.004215

[b21] Zhao Y., Zhang Y. & Wang Q. Optimization of Slow Light in Slotted Photonic Crystal Waveguide With Liquid Infiltration. J. Light. Technol. 31, 2448–2454 (2013).

[b22] Caer C., Le Roux X. & Cassan E. Enhanced localization of light in slow wave slot photonic crystal waveguides. Opt. Lett. 37, 3660–3662 (2012). 22940982 10.1364/OL.37.003660

[b23] Hou J., Gao D., Wu H., Hao R. & Zhou Z. Flat Band Slow Light in Symmetric Line Defect Photonic Crystal Waveguides. IEEE Photonics Technol. Lett. 21, 1571–1573 (2009).

[b24] Tang J. *et al.* Wideband and Low Dispersion Slow Light in Lattice-Shifted Photonic Crystal Waveguides. J. Light. Technol. 31, 3188–3194 (2013).

[b25] Ma J. & Jiang C. Demonstration of Ultraslow Modes in Asymmetric Line-Defect Photonic Crystal Waveguides. IEEE Photonics Technol. Lett. 20, 1237–1239 (2008).

